# Competition of Several Energy-Transport Initiation Mechanisms Defines the Ballistic Transport Speed

**DOI:** 10.1021/acs.jpcb.1c03986

**Published:** 2021-06-29

**Authors:** Sithara U. Nawagamuwage, Layla N. Qasim, Xiao Zhou, Tammy X. Leong, Igor V. Parshin, Janarthanan Jayawickramarajah, Alexander L. Burin, Igor V. Rubtsov

**Affiliations:** Department of Chemistry, Tulane University, New Orleans, Louisiana 70118, United States

## Abstract

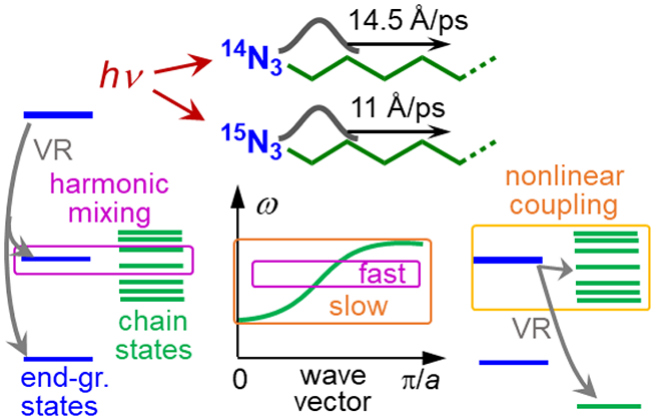

The ballistic regime of vibrational energy transport in oligomeric molecular chains occurs with a constant, often high, transport speed and high efficiency. Such a transport regime can be initiated by exciting a chain end group with a mid-infrared (IR) photon. To better understand the wavepacket formation process, two chemically identical end groups, azido groups with normal, ^14^N_3_-, and isotopically substituted, ^15^N_3_-, nitrogen atoms, were tested for wavepacket initiation in compounds with alkyl chains of *n* = 5, 10, and 15 methylene units terminated with a carboxylic acid (-a) group, denoted as ^14^N_3_C*n*-a and ^15^N_3_C*n*-a. The transport was initiated by exciting the azido moiety stretching mode, the ν_N≡N_ tag, at 2100 cm^–1^ (^14^N_3_C*n*-a) or 2031 cm^–1^ (^15^N_3_C*n*-a). Opposite to the expectation, the ballistic transport speed was found to decrease upon ^14^N_3_ → ^15^N_3_ isotope editing. Three mechanisms of the transport initiation of a vibrational wavepacket are described and analyzed. The first mechanism involves the direct formation of a wavepacket via excitation with IR photons of several strong Fermi resonances of the tag mode with the ν_N=N_ + ν_N–C_ combination state while each of the combination state components is mixed with delocalized chain states. The second mechanism relies on the vibrational relaxation of an end-group-localized tag into a mostly localized end-group state that is strongly coupled to multiple delocalized states of a chain band. Harmonic mixing of ν_N=N_ of the azido group with CH_2_ wagging states of the chain permits a wavepacket formation within a portion of the wagging band, suggesting a fast transport speed. The third mechanism involves the vibrational relaxation of an end-group-localized mode into chain states. Two such pathways were found for the ν_N≡N_ initiation: The ν_N=N_ mode relaxes efficiently into the twisting band states and low-frequency acoustic modes, and the ν_N–C_ mode relaxes into the rocking band states and low-frequency acoustic modes. The contributions of the three initiation mechanisms in the ballistic energy transport initiated by ν_N≡N_ tag are quantitatively evaluated and related to the experiment. We conclude that the third mechanism dominates the transport in alkane chains of 5–15 methylene units initiated with the ν_N≡N_ tag and the wavepacket generated predominantly at the CH_2_ twisting band. The isotope effect of the transport speed is attributed to a larger contribution of the faster wavepackets for ^14^N_3_C*n*-a or to the different breadth of the wavepacket within the twisting band. The study offers a systematic description of different transport initiation mechanisms and discusses the requirements and features of each mechanism. Such analysis will be useful for designing novel materials for energy management.

## 1 Introduction

Ballistic energy transport via acoustic phonons is prevalent in ordered materials such as crystals and can occur to large distances. The amount of energy transferred via wavepackets made of acoustic phonons is limited by the thermal energy, *k*_B_*T*, which is too small to significantly influence chemical processes. In recent studies, ballistic energy transport via covalent bonds of linear oligomeric chains was discovered to occur via optical chain bands, thus delivering substantially larger energy quanta to distances exceeding 60 Å^1−4^ and featuring much higher efficiency compared with that in diffusive energy transport.^5−8^ Oligomeric chains feature a range of chain bands differing in energy and bandwidth. The bandwidth of a chain band determines the mean group velocity of the wavepacket supported by the band.^9^ The ballistic through-chain transport was initiated via excitation with a mid-IR photon, a vibrational mode at the end group, which then transferred its energy into the chain, initiating the transport. The energies of the end-group modes tested for ballistic transport range from 2100 cm^–1^ (azido group stretch)^1^ to 1650–1750 cm^–1^ (carbonyl groups in carboxylic acid, ester, succinimide ester, and amide),^9,10^ 1500 cm^–1^ (amide II mode of an amide),^10^ and ∼1300 cm^–1^ (azido group stretch).^9^ It was found that the selection of the end-group mode used to initiate the transport determines which chain band transfers energy, thus determining the transport speed and efficiency. For example, ballistic transport via alkane chains, initiated by an azido-group stretching mode at 2100 cm^–1^, occurs with a speed of 14.5 Å/ps (1.45 km/s), whereas the speed is 8.0 Å/ps when initiated by a carbonyl stretching mode at 1710–1820 cm^–1^^9^ and only 4.2 Å/ps when initiated with the amide-I mode of an amide end group.^10^ Previous reports by the Troe^11,12^ and Dlott^13,14^ groups indicated the presence of ballistic transport via alkane chains.

Although ballistic transport was reported via several oligomeric chains, PEG,^15^ alkane,^9,12^ perfluoroalkane,^16^ and *p*-phenylene,^17^ the transport initiation mechanisms were not discussed systematically. At the same time, the central role of the transport initiation process became apparent, as the end-group properties define which band or portion of the band is involved in the through-chain transport, thus defining the transport speed and efficiency. This study is inspired by the interest in designing molecular systems with faster and more efficient energy transport. Such systems have high potential as novel materials suitable for energy management, for delivering energy to remote regions, including energy transport against a thermal gradient, and as materials for molecular electronics. The energy quanta delivered via optical chain bands are large enough to be used for remotely initiating low-barrier chemical reactions. Approaches to direct and alternate ballistic transport by external stimuli^10,17^ may result in creating energy-transport switches.

Instead of changing the nature of the end group, drastically changing its properties, in this study, we vary the frequency of the end-group vibrational mode via isotope editing of the end-group atoms, ^14^N_3_– → ^15^N_3_–, targeting different portions of the alkane chain bands and thus expecting different transport speeds. Using the previously reported dispersion curves for alkane chains (Figure S2A in the Supporting Information (SI)),^9^ a higher transport speed was expected for the ^15^N_3_– initiation.

The Article reports the experimental results of energy transport via linear alkyl chains of 5, 10, and 15 methylene groups terminated with an isotopically edited azido group and a carboxylic acid group, ^15^N_3_C*n*-a (Section 3.1). The experiments were performed using relaxation-assisted two-dimensional infrared (RA 2DIR) spectroscopy, where the excess energy arrival from the initially excited end-group mode (tag) to the site of the end group at the opposite chain end (reporter) was recorded as a time dependence of the tag-reporter cross-peak amplitude.^7^ A systematic description of the ballistic transport initiation mechanisms is given in Section 3.2. Sections 3.3–3.6 discuss different mechanisms of transport initiation in N_3_C*n*-a, describing vibrational relaxation pathways of the tag and its relaxation daughter modes and the coupling strength among the end-group and chain states. In the Discussion section, we compare different mechanisms of transport initiation, offering strategies to design compounds to achieve high efficiency of energy delivery and high transport speed.

## 2 Experimental Details

### 2.1 2DIR Measurements

A comprehensive description of a fully automated dual-frequency three-pulse echo 2DIR instrument with heterodyne detection is presented elsewhere.^18^ In brief, a Ti:sapphire laser producing 1.5 W power at 1 kHz repetition rate, 800 nm wavelength, and 80 fs pulse duration (Libra, Coherent) was used to pump a computer-controlled dual optical parametric amplifier (OPA, Palitra-duo, Quantronix). Two pairs of signal and idler pulses generated by the OPA were directed to two computer-controlled difference frequency generation (DFG) units (NIR Quantronix) to generate mid-IR pulses tunable in the frequency range from 500 to 5000 cm^–1^, featuring a pulse energy ranging from 1.0 to 10 μJ. A fully automated 2DIR instrument features a sensitivity of better than 10^–4^ cm^–1^ in measured anharmonicities, which is achieved by a combination of a closed-loop phase stabilization of better than 70 as, phase cycling, and spectral interferometry. The automatic frequency tuning from 800 to 4000 cm^–1^ is achieved by implementing a mid-IR beam direction stabilization schematic (<50 μrad deviations)^19^ and a schematic for setting the phase-matching geometry for mid-IR beams at the sample.^18^ The spectral width of the mid-IR pulses was ∼150 cm^–1^, and the instrument response function was ∼140 fs. The 2DIR measurements were performed by scanning the delay between the first two mid-IR pulses τ of the same DFG unit at a fixed waiting time *T*, which is the delay between the second and third pulses, and recording the heterodyned spectrum in the frequency range of interest (λ → ω_*t*_) for every τ. Fourier transformation along τ results in the ω_τ_ axis in the 2DIR spectrum, shown as the ordinate. A typical 2DIR spectrum contained ∼250 points along the τ direction and took 1–3 min to acquire. For the RA 2DIR measurements, the 2DIR spectra were recorded for each waiting time, which was scanned with nonconstant delay steps ranging from 100 fs at small waiting times up to 5 ps at large waiting times. Typical waiting-time dependences contain 40–50 points along *T*, which take 1 to 2 h to acquire.

### 2.2 Sample Preparation

**Figure 1:**
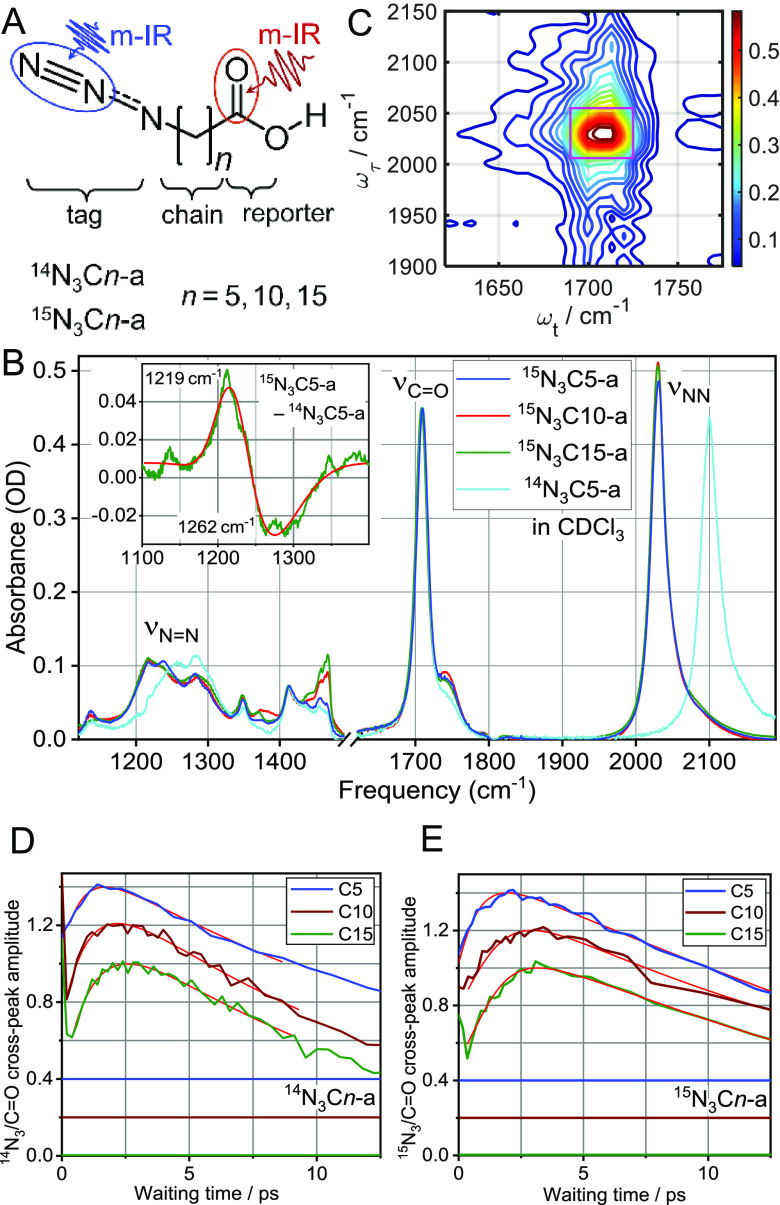
(A) Molecular structure of the used compounds. (B) Linear absorption spectra of the ^15^N_3_C5-a, ^15^N_3_C10-a, ^15^N_3_C15-a, and ^14^N_3_C5-a in CDCl_3_, normalized for the carbonyl peak at ν_C=O_ at ca. 1709 cm^–1^. Two azido-group vibrational modes denoted as ν_N≡N_ (2100 cm^–1^ in ^14^N_3_C*n*-a and 2031 cm^–1^ in ^15^N_3_C*n*-a) and ν_N=N_ (∼1250 cm^–1^) are labeled. The inset shows the ν_N=N_ difference spectrum ^15^N_3_C5-a – ^14^N_3_C5-a and its fit with two Gaussian functions. (C) 2DIR spectrum showing the ν_N≡N_/ν_C=O_ cross-peak for ^15^N_3_C15-a at *T* = 3.1 ps. (D,E) Waiting-time dependences for the ν_N≡N_/ν_C=O_ cross-peak for (D) ^14^N_3_C*n*-a^2^ and (E) ^15^N_3_C*n*-a for *n* = 5, 10, and 15. Notice that the graphs are spaced vertically, and zero horizontal lines are shown with matching colors. Fitting curves with an asymmetric double-sigmoidal function are shown with red lines.

A series of compounds featuring an isotopically edited azido end group, an alkyl chain of three lengths of *n* = 5, 10, and 15, and a carboxylic-acid-terminating moiety, denoted as ^15^N_3_C*n*-a (Figure 1A), were prepared according to the procedure previously reported for ^14^N_3_C*n*-a, except isotopically labeled sodium azide (Na^15^N_3_, 97% ^15^N, Berry & Associates/ICON Isotopes) was used as the reagent.^2^ Fourier transform infrared (FTIR) and 2DIR measurements of N_3_C*n*-a compounds dissolved in CDCl_3_ were performed in a sample cell made of 1 mm thick CaF_2_ windows and a 50 μm Teflon spacer at room temperature, 22 ± 0.5 °C.

### 2.3 Density Functional Theory Calculations

Density functional theory (DFT) calculations were performed using the Gaussian 09 suite. Geometry optimization, vibrational mode analysis, and anharmonicity calculations were executed using the B3LYP functional and the 6-311+G(d,p) basis set in dichloromethane (DCM) solvent described with a polarized continuum model (PCM) method. Vibrational relaxation pathways of the end-group states were computed using the theoretical approach reported in refs (20 and 21). The method uses DFT-computed anharmonic coupling constants of an isolated molecule to compute third-order relaxation pathways. The relaxation pathways from several excited azido-group states were performed for compounds with *n* = 1 and 5 and terminated with a hydrogen atom of 2000 amu instead of the carboxylic acid moiety, denoted as ^15^N_3_C*n* and ^14^N_3_C*n*.

Vibrational couplings between the modes of the azido moiety and chain states were evaluated for both ^14^N_3_C*n* and ^15^N_3_C*n* compounds with *n* = 1 and 5 using the previously reported approach where the atomic masses of the end-group atoms are smoothly changed, causing the end-group frequency change, and the Hessian matrix is diagonalized for each mass point.^9^ As the end-group frequency passes the resonance with a chain state, a frequency jump occurs that is equal to 2β, where β is the interaction energy of the end-group and the chain state.^9^

## 3 Results

### 3.1 2DIR Measurements of the Energy Transport

Both high-frequency modes of the azido moiety, denoted as ν_N≡N_ and ν_N=N_, are significantly affected by the ^14^N_3_– → ^15^N_3_– isotope editing. (See Section S1 in the SI.) The frequency of the ν_N≡N_ mode, found at 2100 cm^–1^ in ^14^N_3_C*n*-a, is red-shifted in ^15^N_3_C*n*-a by 69.2 cm^–1^ (Figure 1B). The frequency of the N=N mode, found at ca. 1270 cm^–1^ in ^14^N_3_C*n*-a, is also red-shifted in ^15^N_3_C*n*-a by ca. 45 cm^–1^ (Figure 1B, inset).

**Figure 2:**
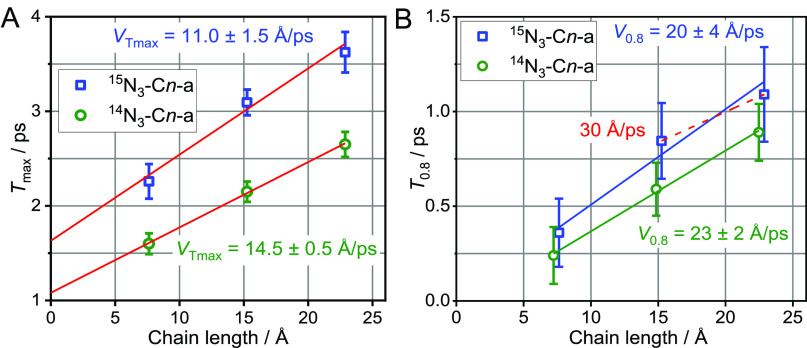
(A) *T*_max_ and (B) *T*_0.8_ values as a function of chain length for the two series of compounds, ^14^N_3_C*n*-a and ^15^N_3_C*n*-a, with *n* = 5, 10, and 15. Linear fit lines are shown in red, and the corresponding transport speeds obtained from the fits are shown in data-matching colors.

2DIR cross-peak spectra, focusing at the ν_N≡N_/ν_C=O_ cross-peak, were measured for ^15^N_3_C*n*-a by tuning the first two IR pulses to excite the ν_N≡N_ tag mode at 2031 cm^–1^ and the third and local oscillator (LO) pulses to access the ν_C=O_ reporter mode at 1709 cm^–1^ (Figure 1C). The cross-peak spectra were measured for a range of waiting times, *T*, and waiting-time kinetics were constructed by integrating the 2DIR spectra over the cross-peak area and plotting the resulting amplitude as a function of the waiting time (Figure 1E). The results for ^14^N_3_C*n*-a are shown in Figure 1D, reported in ref (2). The traces show a rise with an increase in the waiting time associated with the energy transport from the tag to the reporter site. The maximum in the kinetic trace, occurring at *T*_max_, corresponds to the maximum energy delivered; the following decrease in the cross-peak amplitude is associated with the cooling of the reporter site in the solvent, occurring with the characteristic time of ca. 14 ps. To determine the *T*_max_ value for each compound, the traces were fitted with an asymmetric double-sigmoidal function. (See Figure S1 in the SI for details.) Mean *T*_max_ values were determined by averaging three measurements per compound. (See Section S2 in the SI.) The resulting *T*_max_ values were plotted as a function of the through-bond chain length, determined as a sum of all C–C bond lengths of the chain (Figure 2A). The dependence was fitted with a linear function, and the transport speed, *V*, was evaluated as an inverse slope of the fit function. The energy-transport speed along the chain in ^15^N_3_C*n*-a was found to be 11.0 ± 1.5 Å/ps, which is slower than the 14.5 ± 0.5 Å/ps reported for ^14^N_3_C*n*-a (Figure 1D).^2^

Note that this result is opposite to the prediction of a higher speed with ^15^N_3_– transport initiation. The expectation was based on the calculations in which ν_N=N_, a daughter mode of ν_N≡N_ relaxation, matched the portion of the dispersion curves of both the CH_2_ wagging and twisting chain bands, which feature higher group velocities. (See Section S3 in the SI.)

It is also notable that longer *T*_max_ times are observed for ^15^N_3_C*n*-a compared with ^14^N_3_C*n*-a (Figure 2A); the *T*_max_ values obtained from linear extrapolation to zero chain length differ by ca. 0.5 ps. The increase in *T*_max_ suggests that the energy injection into the chain occurs slower in ^15^N_3_C*n*-a.

The presence of different wavepackets featuring different speeds is expected for both isotopes. Faster moving wavepackets contribute the most at the rising portion of the waiting-time trace. To inspect the contributions of faster wavepackets, the transport speed was also evaluated based on the initial portion of the waiting-time traces, the *T*_0.8_ values, determined as the time in which the trace reached the 0.8 fraction of its maximum amplitude (Figure 2B).^17,22^ The obtained speed is higher than the *T*_max_-based speed (Figure 2B inset) by a factor of 1.6 to 1.8.

A theoretical analysis of the energy injection process was performed for both end groups. The analysis focused on the relaxation channels of the tag (ν_N≡N_) and its relaxation daughter modes (ν_N=N_ and ν_N–C_), coupling the daughter modes and the chain states, and Fermi resonances (FRs) of the tag. We first present a general description of different mechanisms of wavepacket initiation.

### 3.2 Mechanisms of Wavepacket Formation

**Figure 3:**
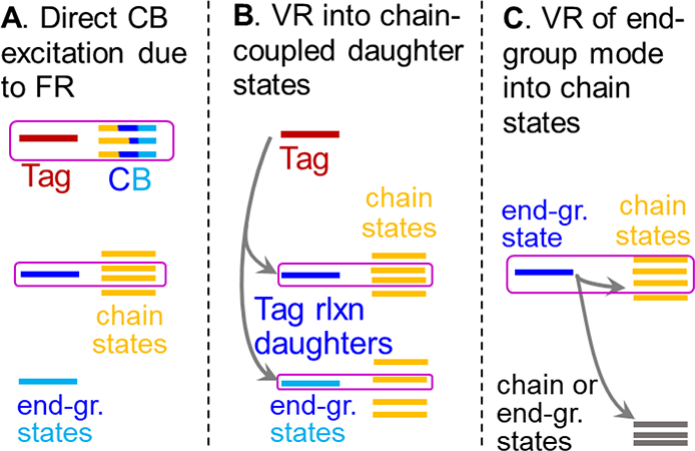
Mechanisms of ballistic transport initiation by an excited end-group-localized vibrational mode. Magenta boxes indicate the couplings required for wavepacket initiation.

Wavepacket initiation by an excited end-group-localized mode requires energy transfer from the end group to the chain states. Excitation of a single-exciton coherent superposition of chain states, a wavepacket, can occur directly from the excited tag mode or from the daughter modes of the tag relaxation. Different mechanisms of wavepacket excitation rely on different extents and orders of interaction between the end-group modes and the chain states, as shown in Figure 3. Without losing generality, we consider the tag that is end-group-localized and frequency-isolated, so it is not harmonically mixed with any chain state. Nevertheless, the tag mode can be anharmonically coupled to other states via FRs. If a strong FR occurs among the tag and a combination band involving chain-band states, then the combination band will gain IR intensity through intensity borrowing from the tag (Figure 3A). It is possible to have multiple FRs involving a range of chain states, as reported for the azido end group.^23^ Such combination bands can be directly excited with a photon and can, under certain conditions, form a wavepacket within the chain states.

Another mechanism of wavepacket formation involves vibrational relaxation of the tag into daughter states, at least one of which is strongly (harmonically) coupled to the chain states, β_end gr._ (Figure 3B). Strong coupling among a daughter mode and a few chain states results in their mixing. Vibrational relaxation, local in nature, excites, in this case, a superposition of delocalized chain states, such that they involve local motion at the sites nearest to the end group, a wavepacket. The spectral width of the wavepacket is defined by the coupling strength between the end-group daughter mode and the chain state localized at the adjacent methylene site, β_end gr./ch._, in relation to the coupling within the chain, β_ch._. If the coupling strength is comparable to or larger than the chain bandwidth, β_end gr._ ≥ β_ch._, then all states of the band will be involved in the wavepacket. The transport speed of such a wavepacket corresponds to the mean speed of the chain band. If β_end gr._ < β_ch._, only the chain states that are resonant with the end-group mode will participate in the wavepacket. Such a wavepacket will feature a group velocity that is characteristic of a specific portion on the chain band dispersion curve at the frequency of the chain state, not the mean speed supported by the whole band. The spatial size of any wavepacket is defined by the number of chain states involved in the superposition. For the cases with smaller β_end gr./ch._, longer chains are required to achieve a spatially sharp wavepacket.

Another mechanism of wavepacket formation involves the vibrational relaxation of the end-group mode into chain states (Figure 3C). The local nature of the relaxation process is derived from the spatial proximity of the involved states for strong coupling.^24−26^ Therefore, strong anharmonic coupling of the end-group mode and the local mode at the first chain site is require for efficient relaxation. Anharmonically driven relaxation will excite a local chain mode, which represents a wavepacket on the basis of delocalized chain states. The strength of the anharmonic coupling determines the spectral width of the wavepacket and its spatial sharpness, similar to case B.

In the next section, we will consider the transport initiation with ν_N≡N_ in ^14^N_3_C*n*-a and ^15^N_3_C*n*-a to identify which mechanism is contributing the most. To identify the differences and similarities in the transport, computational results are presented for compounds with both isotopes. First, we consider a possibility of mechanism B, inspecting the relaxation pathways of the tag and the extent to which the daughter modes of the tag relaxation involve the chain states.

### 3.3 Vibrational Relaxation Pathways for the ν_N≡N_ Tag

**Figure 4:**
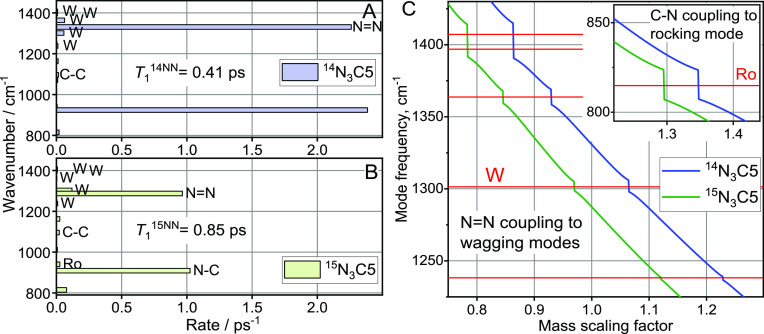
Relaxation pathways for the tag mode, ν_N≡N_, computed for (A) ^14^N_3_C5 and (B) ^15^N_3_C5. (C) ν_N=N_ mode frequency of ^14^N_3_C5 (blue) and ^15^N_3_C5 (green) as a function of the mass scaling factor for the N atoms of the azido group. Horizontal red lines show the frequencies of the wagging chain states. The inset shows the ν_N–C_ mode frequency as a function of the mass scaling factor, indicating its coupling to the rocking state at 815 cm^–1^.

We computed the rates of the intramolecular vibrational energy redistribution (IVR) pathways using a recently developed theoretical approach.^20,21^ To determine the vibrational frequencies and anharmonic force constants, DFT anharmonic computations were performed using the Gaussian-09 suite^27^ for the ^14/15^N_3_-(CH_2_)_5_-^2000^H compounds, denoted as ^14^N_3_C5 and ^15^N_3_C5, where a large mass for the chain-terminating hydrogen was used to decouple its motion from other motions in the compounds. In agreement with previous reports for ^14^N_3_– initiation,^2,23^ the ν_N≡N_ relaxation predominantly populates a combination band of modes ν_N=N_ and ν_N–C_ (Figure 4A,B), which accounts for >90% of all relaxation channels for both isotopes. To efficiently create a wavepacket in the chain upon the tag relaxation, the relaxation daughter states need to be harmonically mixed with more than one chain state of the same chain band (mechanism B). The ν_N=N_ daughter state frequency matches the CH_2_ wagging (W) and twisting (Tw) chain bands (Figure S2 in the SI), whereas ν_N–C_ is located within the rocking (Ro) band (Figure S4 in the SI).

To evaluate the coupling strength of the ν_N=N_ mode with the wagging and twisting modes of the chain, we scanned the masses of the nitrogen atoms of the azido moiety in increments of 0.01 amu and computed the normal modes for every mass using the Hessian matrix obtained via DFT normal-mode analysis.^9^ When, as a result of mass change, the frequency of ν_N=N_ passes that of the chain state, W or Tw, a jump of the mode predominantly residing on the azido group (ν_N=N_) occurs. The magnitude of the frequency jump is equal to 2β_N=N/W_ or 2β_N=N/Tw_, where β is the respective interaction energy. The computations performed for N_3_C1 resulted in β_14N=N/W_ = 10 cm^–1^, β_15N=N/W_ = 8.4 cm^–1^, and β_N=N/Tw_ < 0.1 cm^–1^ for both isotopes. Similar results were obtained for N_3_C5, although the observed coupling was different for different wagging states (Figure 4C) correlating with a contribution of the first methylene site in different wagging normal modes; this contribution is the highest at ca. 1/√*n* for the normal modes featuring the highest wave vectors, where *n* is the number of sites. Note that equal site contributions require cyclic boundary conditions.

Therefore, ν_N=N_ is harmonically mixed with wagging modes but only within a rather narrow resonance window of about ±2β_N=N/W_. The pattern of ν_N≡N_ relaxation pathways confirms the result: In addition to the relaxation pathway to ν_N=N_ (Figure 4A), there is a pathway in ^15^N_3_C5 to a single wagging state, which is shifted by ca. 15 cm^–1^ from ν_N=N_, and two pathways in ^15^N_3_C5 to wagging states that are shifted by ca. 25 cm^–1^ from ν_N=N_. These pathways gain their efficiencies due to the mixing of ν_N=N_ with formalwagging states of the chain. Because the site energy gap is larger than β_N=N/W_, the mixing is partial. The other wagging states of the chain are further away from ν_N=N_ and do not feature significant ν_N=N_ contributions. As a result of the small density of chain states in N_3_C5, the ν_N≡N_ relaxation mostly populates the end-group-localized ν_N=N_, although a small amplitude wavepacket is also created on the wagging states. The chain-state density increases with the increase in chain length. For the efficient formation of the wavepacket, at least two wagging states need to be found within the 2β_N=N/W_ ≈ 20 cm^–1^ window at the frequencies around ν_N=N_. To satisfy this requirement, the chain length should be ca. 22 and 19 units long for normal and isotopically substituted compounds. Even for the longest experimentally assessed chain of N_3_C15-a, two wagging states will not be efficiently excited via mechanism B, suggesting that the influence of such a wavepacket will likely be small for the overall energy-transport process. A rather high speed is expected for such a wavepacket, reaching 40 Å/ps for ^14^N_3_C5, as the fastest portion of the wagging band will be involved, not the whole band. Essentially the same speed is predicted for ^15^N_3_C5 for the wagging band constructed of either harmonic or anharmonic chain states. (See Section S3 in the SI.)

The coupling between ν_N–C_ and the rocking band states, β_N–C/Ro_, evaluated in a similar way to β_N=N/W_, was found to be ∼8 cm^–1^ (Figure 4C, inset). The density of the rocking states at ca. 900 cm^–1^ is the smallest within the band, corresponding to the highest group velocity of the rocking band, exceeding 65 Å/ps. To have two rocking states separated by 2β_N–C/Ro_ ≈ 16 cm^–1^ for their efficient mixing with ν_N–C_, the chain should consist of 44 methylene units. The longest experimentally assessed chain, N_3_C15-a, is almost three times shorter, which allows us to conclude that the formation of a wavepacket on rocking states via mechanism B in negligible for these chains. Therefore, the vibrational relaxation of ν_N≡N_ remains mostly local to the azido moiety and will not efficiently create a wavepacket on wagging, twisting, or rocking states via mechanism B.

Next, we consider relaxation pathways of the first-tier daughters, ν_N=N_ and ν_N–C_, and the possibility of wavepacket formation via their relaxation into chain states (mechanism C).

### 3.4 Vibrational Relaxation Pathways of ν_N=N_

The ν_N=N_ mode lifetime, computed for both isotopes in N_3_C5, is very short at 0.20 (^14^N) and 0.18 ps (^15^N). Three groups of daughter modes account for 95% of all pathways: all CH_2_ twisting modes (36.5 rate percent for ^14^N and 50 rate percent for ^15^N), NNN bending mode overtone, δ_NNN_ (35% for ^14^N and 29% for ^15^N), and ν_N–C_ (23% for ^14^N and 17% for ^15^N) (Figure S5 in the SI). The δ_NNN_ mode frequency, ∼600 cm^–1^, is too low to populate any optical chain bands in the following relaxation process. The excitation of the ν_N–C_ mode will be considered in the next section, as ν_N–C_ is also a daughter mode of ν_N≡N_ relaxation.

**Figure 5:**
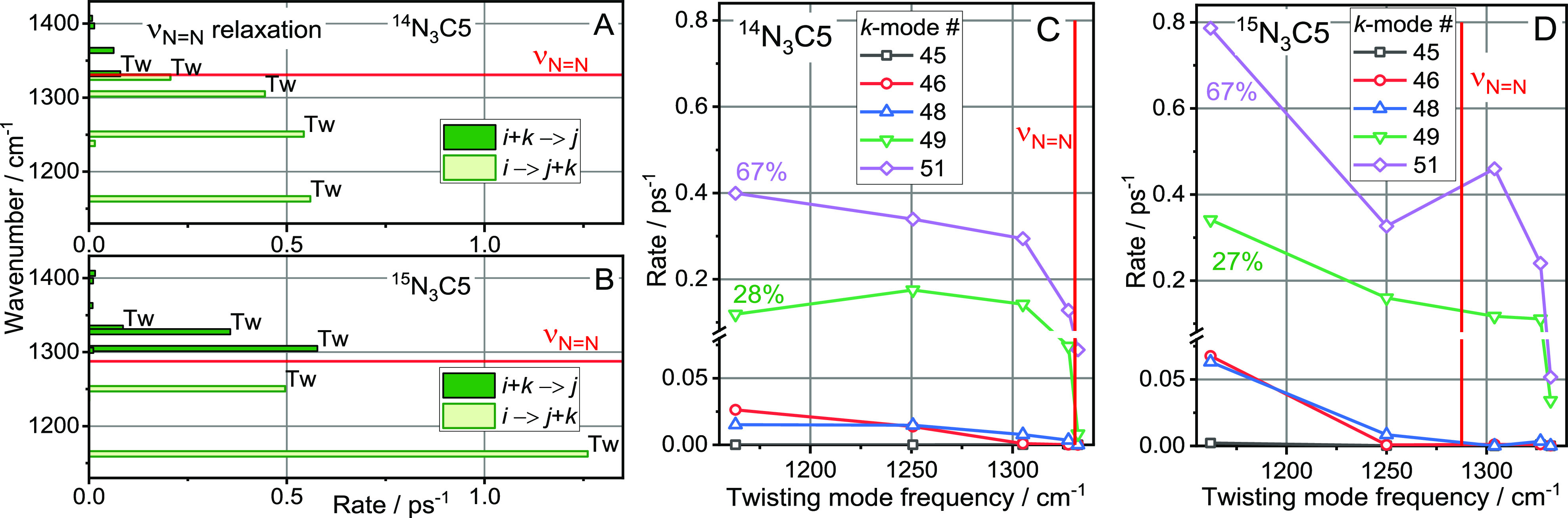
Relaxation pathways of the ν_N=N_ populating twisting modes of the chain, computed for (A) ^14^N_3_C5 and (B) ^15^N_3_C5. (See the complete set of data in Figure S5 in the SI.) Red horizontal lines show the position of ν_N=N_ for each isotope. (C,D) Individual relaxation channels populating the twisting band states for *i* → *j* + *k* and *i* + *k* → *j* processes, where *i* denotes ν_N=N_, *j*(*k*) denotes one of the twisting modes, and *k* is a low-frequency mode involved in the IVR step. The points for the same *k* values are connected. The frequencies of the *k* modes showing significant contributions are 25 (#51), 60 (#49), 81 (#48), 135 (#46), and 148 cm^–1^ (#45).

The ν_N=N_ mode relaxation pathways populate multiple twisting chain states in ^14^N_3_C5 and ^15^N_3_C5, as shown in Figure 5A,B. Red horizontal lines show the location of the ν_N=N_ states for respective isotopes. Whereas all twisting states feature appreciable rates and the wavepacket formation seems possible, an additional requirement has to be satisfied to form a wavepacket rather than populating individual states with no specific phase relations characteristic of a wavepacket. The third-order relaxation process involves a parent mode (ν_N=N_, mode *i*), one of the twisting modes (*j*), and a low-frequency mode (*k*). The relaxation process of mode *i* will create a wavepacket only if multiple chain states *j* become populated for the same mode *k*. On the contrary, if different *k* states are involved for different chain states *j*, then individual chain states will be excited, not a wavepacket. Five low-frequency *k* modes are involved in the relaxation process. Two of them, modes #49 and #51, result in the strongest rates across all twisting states (Figure 5C,D). These two decay channels are characterized by a sufficiently strong third-order interaction and involve all twisting chain states spread over a range of >160 cm^–1^ in frequency. Relaxation channels involving both low-frequency modes will generate wavepackets in the twisting bands. About 95% of all relaxation channels populate twisting-band states for both ^14^N_3_C5 and ^15^N_3_C5 (Figure 5C,D), so the wavepacket formation is efficient. Essentially the whole twisting band is involved in the wavepacket, so the transport speed can be estimated as the mean group velocity for the band: ,^9^ computed at 10.4 Å/ps (Figure S2 and Table S2 in the SI).

### 3.5 Vibrational Relaxation Pathways for ν_N–C_

**Figure 6:**
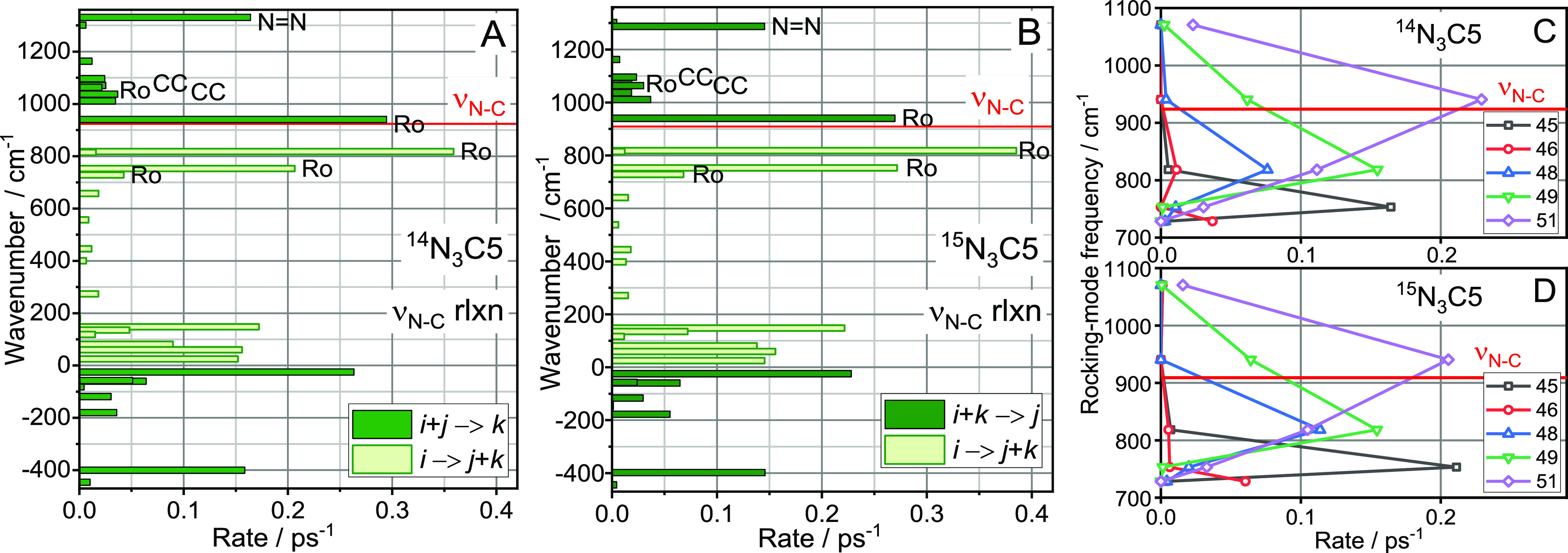
All relaxation pathways of the ν_N–C_ mode for (A) ^14^N_3_C5 and (B) ^15^N_3_C5 (B). The majority of the pathways populate rocking modes of the chain (Ro). Red horizontal lines show the position of ν_N–C_ for each isotope. (C,D) Individual relaxation channels populating the rocking band states for *i* → *j* + *k* and *i* + *k* → *j* processes where *i* denotes ν_N–C_, *j*(*k*) denotes one of the rocking modes, and *k* is a low-frequency mode involved in the IVR step. Points for the same *k* values are connected. The frequencies of the *k* modes showing significant contributions are 25 (#51), 60 (#49), 81 (#48), 135 (#46), and 148 cm^–1^ (#45).

The ν_N–C_ mode lifetime was computed at 0.78 ps for ^14^N_3_C5 and 0.75 ps for ^15^N_3_C5. The relaxation pathways predominantly involve the rocking states of the chain (Figure 6A,B), which account for 72.3 and 76.1 rate percent, respectively. Note that all rocking states are significantly populated, although not evenly. To check if a common relaxation process exists for each rocking mode and the same low-frequency mode, we analyzed individual third-order pathways of ν_N–C_ relaxation, shown in Figure 6C,D. Five low-frequency modes (*k* = 45, 46, 48, 49, 51) are involved in the *i* → *j* + *k* and *i* + *k* → *j* processes, where *i* denotes ν_N–C_ and *j* is one of the rocking band states. The pathways involving *k* modes 45, 46, and 48 populate a single rocking state and cannot form a wavepacket. *k* modes 49 and 51 populate more than one rocking states and will form a wavepacket-localized chain excitation on the azido side.

The speed of such a wavepacket is expected to be extremely high at ca. 67 Å/ps, as the fast portion of the rocking band is involved (Table S2). Interestingly, the yield of the wavepacket formation with modes 51 and 49 is rather high, with 57 and 52% of all relaxation channels into rocking modes for ^14^N_3_C5 and ^15^N_3_C5, respectively. Despite the rather high predicted efficiency, such high speeds have not been clearly observed experimentally, possibly due to the following reasons: (1) A large spatial size of the wavepacket is expected as a small number of chain states is involved. A large wavepacket size will likely reduce the efficiency of its relaxation to the reporter site as its participation at the last chain site is diminished by broadening. Note, however, that the quality of the wavepacket will increase for longer chains, as more states will be covered by the strength of the involved nonlinear interaction, which is ca. 50 cm^–1^. The rocking-state frequencies are low, which could result in their faster relaxation, although this is not confirmed experimentally. (2) It is difficult to experimentally tell apart such fast transport from a nonresonant signal appearing at *T* ≈ 0 ps, especially if slower but more efficient transport pathways are present. Note that the time to pass a C15 chain for such a wavepacket is 0.35 ps, which is only two times longer than the instrument response time (Figure S6B in the SI) and is much smaller than the experimental *T*_max_ and even *T*_0.8_ values (Figure 2B). The difference in the speeds determined from *T*_max_ and *T*_0.8_ indicates the contributions of faster transport processes, which likely include the transport via the rocking band (Figure 2B). Note that other chain bands, such as ν_C–C_ and scissoring, are found to be much less populated via the first two tiers of ν_N≡N_ relaxation and are not expected to participate in the energy transport initiated by ν_N≡N_.

### 3.6 Wavepacket Formation via Direct Excitation of ν_N≡N_ Fermi Resonances (Mechanism A)

The FR between the ν_N≡N_ fundamental and the combination band of ν_N=N_ and ν_N–C_ modes is strong, resulting in significant intensity borrowing. Moreover, the FRs were shown to be a major reason for a large ν_N≡N_ transition width, observed even in nonpolar solvents.^23^ Both states of the combination state are involved in mixing with the chain states, wagging for ν_N=N_ and rocking for ν_N–C_, resulting in more than one FR. The mixing increases with the increase in chain length, and the number of FRs is expected to increase. The coupling of the ν_N≡N_ fundamental and the ν_N=N_ + ν_N–C_ combination band, defined from anharmonic calculations with Gaussian 09, is ca. 30 cm^–1^. This value is about three times larger than β_N=N/W_ or β_N–C/Ro_, thus providing no limitations for the mixing. Therefore, the mixing of the ν_N=N_ and ν_N–C_ states with the chain states will determine the number of FRs for the tag. As long as the mixing with chain states occurs, the FR will be strong enough to bring IR intensity to combination bands via intensity borrowing. As a result, the wavepacket on wagging and rocking bands directly initiated by absorbing a photon will be required to satisfy the same density of chain-state conditions at ν_N=N_ and ν_N–C_ as the wavepackets following mechanism B. A similarly high transport speed is expected because only a portion of the chain bands will participate in the superposition. The key difference between mechanisms A and B is that a wavepacket via mechanism A is formed without a delay, whereas in mechanism B, the wavepacket is delayed by the lifetime of the tag. Calculations of the FRs, performed for the two isotopes (see Section S5 in the SI), did not reveal differences that could be related to the experimentally measured speeds reported here. Thus mechanism A of wavepacket initiation still requires experimental confirmation.

### 3.7 Classical Modeling of Ballistic Transport

**Figure 7:**
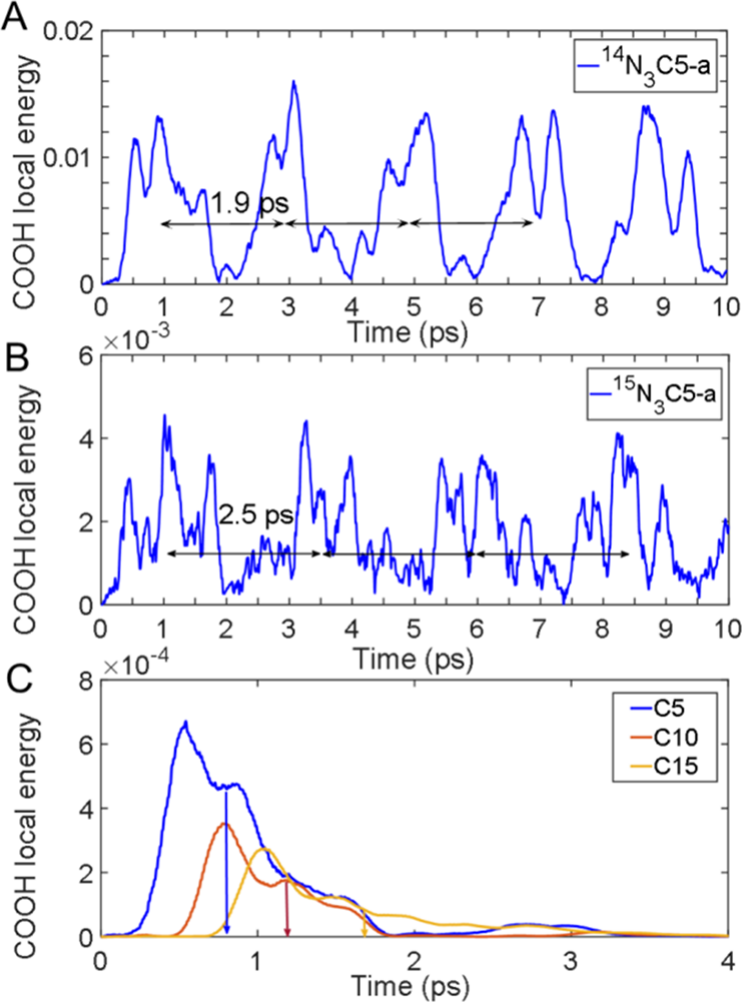
COOH-group local energy versus time for (A) ^14^N_3_C5-a, (B) ^15^N_3_C5-a with infinite damping time, and (C) ^14^N_3_C*n*-a with a damping time of 1.5 ps and *n* = 5, 10, 15. The color-matching arrows show the average arrival times, *t*_D_, calculated using eq S5 in the SI.

Ballistic energy transport in N_3_C*n*-a was modeled by solving classical Newton’s equations using DFT-determined harmonic force constants (Hessian). (See the details in Section S9 in the SI.) Note that harmonic approximation, unsuitable for intramolecular energy redistribution, is expected to work reasonably well for the wavepacket transport. The initial excitation at ca. 1300 cm^–1^ was introduced by exciting a local ν_N=N_ mode, the relaxation daughter of the ν_N≡N_ tag. Energy arrival to the reporter was monitored by summing all local energies of the carboxylic acid end-group atoms. In the absence of energy damping channels, an approximately periodic time dependence was obtained, as the excess energy is readily reflected from the chain ends (Figure 7A). The recurrency period, *t*_R_, corresponding to the energy round trip, is related to the transport speed as *V* = 2*L*/*t*_R_, where the chain length, *L*, was taken as *L* = *n*·*l*_CC_ + *l*_NC_. Here *l*_CC_ and *l*_NC_ are the CC and CN bond lengths, and *n* is the number of methylene units in the chain (*n* = 5, 10, 15).

A comparison of the transport speeds for ^14^N_3_C5-a and ^15^N_3_C5-a (Figure 7A,B) showed that the speed is smaller in ^15^N_3_C5-a for all chain lengths (Table S5). The average speeds of 12 ± 3 and 6 ± 1.5 Å/ps were obtained for ^14^N_3_C*n*-a and ^15^N_3_C*n*-a, respectively. A damping factor, representing total dephasing of the chain states, was introduced to eliminate the energy arrival recurrencies. The transport speed was found to be somewhat affected by the value of the damping factor. For the damping factor of 1.5 ps (Figure 7C), a better match with the experimentally observed speeds was found with 13 and 10 Å/ps for ^14^N_3_C*n*-a and ^15^N_3_C*n*-a, respectively.

## 4 Discussion

**Figure 8:**
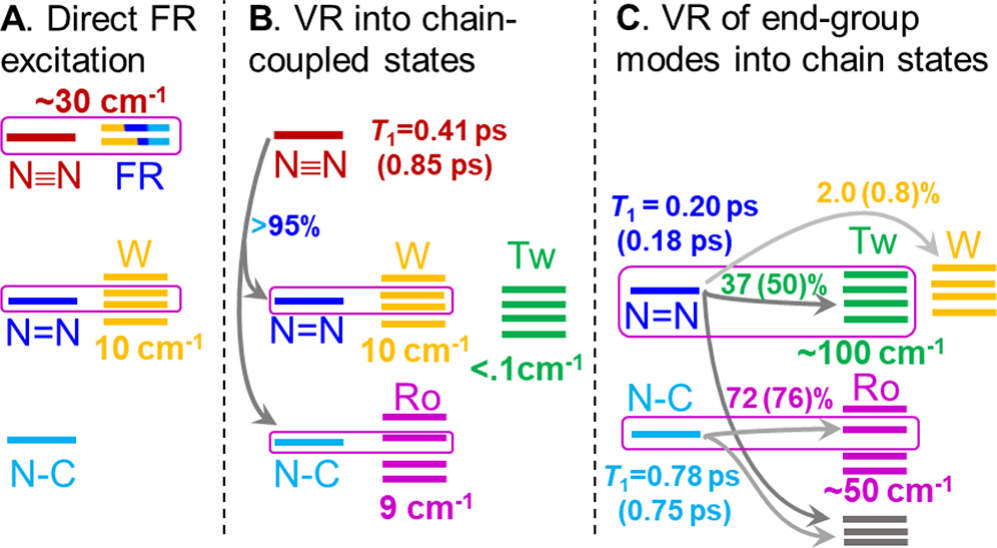
Mechanisms of ballistic transport initiation by the excited ν_N≡N_ end-group mode. The numbers indicate couplings, if in wavenumbers, lifetimes, if in picoseconds, and rate percent populating band states, if in percent. The values in parentheses computed for ^15^N_3_C5 follow the values for ^14^N_3_C5, unless the two values are within a few percent of each other.

Figure 8 summarizes the findings for ballistic transport initiated with ν_N≡N_ in ^14^N_3_- and ^15^N_3_- end groups (values for ^15^N_3_- are shown in parentheses). Mechanism C is found to be contributing the most for both isotopes. The dominant wavepacket propagates via twisting band with the speed of ca. 10 Å/ps for both isotopes. A spatially broad wavepacket at rocking states is expected (mechanism C), propagating with an extremely high speed, exceeding 60 Å/ps. In addition, mechanisms A and B may contribute to ballistic transport via wagging band; the wavepacket is expected to be spatially broad and feature high speed of ca. 36 Å/ps (Section S3 in SI).

^14^N_3_C5 and ^15^N_3_C5 compounds show large similarities in the types of chain bands involved and in the group velocities expected for each band. Two hypotheses are proposed to account for the differences observed in the expriments. First, differences in the contributions of various involved wavepackets for the two isotopes determine the overall outcome—faster *V*_*T*_max__ speed for ^14^N_3_C*n*-a. Indeed, the formation of the dominant wavepacket on twisting band via mechanism C is more efficient for ^15^N_3_C*n*-a (Figure 8C). This wavepacket defines the *V*_*T*_max__ speed in ^15^N_3_C*n*-a. Predicted at 10.4 Å/ps (Table S2 in the SI), the speed well matches the experimental value of 11.5 Å/ps in ^15^N_3_C*n*-a. The formation of such a wavepacket in ^14^N_3_C*n*-a is less efficient (Figure 8C), and faster wavepackets may affect the overall transport more significantly, resulting in faster *T*_max_ and *T*_0.8_ speeds and smaller *T*_max_ values observed experimentally.

Alternatively, the slower transport speed in ^15^N_3_C*n*-a may be due to a lower group velocity of the dominant wavepacket on the twisting band due to a larger involvement of higher energy states of the band. Such a statement sounds counterintuitive, as the ν_N=N_ frequency shifts down in ^15^N_3_C*n*-a. However, as a result of such a shift, the ν_N=N_ relaxation process in ^14^N_3_C*n*-a occurs predominantly downhill (*i* → *j* + *k*), whereas there is a larger uphill contribution, *i* + *k* → *j* channels in ν_N=N_ → Tw relaxation, in ^15^N_3_C*n*-a (Figure 5D). The efficiency of the uphill process linearly depends on the population of mode *k*. Compounds with a larger chain length feature lower frequencies of acoustic modes, which will be thermally populated to larger quantum numbers and offer increased rate contributions in ν_N=N_ → Tw relaxation. At the same time, the twisting states involved in uphill relaxation correspond to the top of the twisting chain band and feature smaller local group velocity. Note that the downhill relaxation channels, *i* → *j* + *k*, are less sensitive to the energy values of modes *k*, so their rates are not expected to change much for compounds with longer chains. We hypothesize that a significantly larger contribution of uphill relaxation channels in ^15^N_3_C5 results in a smaller mean speed of the wavepacket on the twisting band. A significant difference in the ν_N≡N_ lifetime of ca. 0.4 ps was computed for ^14^N_3_C*n*-a (Figure 8A). However, experimentally measured lifetimes for the two isotopes did not support this prediction, showing similar lifetimes for the two isotopes (Figure S6 in the SI).

No significant mixing among ν_N=N_ and the twisting modes was found when the azido group was attached to the chain in an anti conformation, which is energetically favorable over the gauche conformation, but only by ca. 96 cm^–1^. In the gauche conformation of the N_3_ attachment, β_N=N/Tw_ has similar values to β_N=N/W_; both are ca. 10 cm^–1^. Note that the anti kink at the azido-group side of the chain results in mixing of the identities of the delocalized chain states and the inability to assign them as purely wagging or twisting. The treatment of such less regular but still delocalized states will be considered elsewhere. Nevertheless, the mixing of ν_N=N_ is still small enough that (i) longer chains are required to form the wavepacket efficiently and (ii) the expected transport speed corresponds to that of the fast portion of the original wagging and twisting bands, which exceeds the experimentally observed speed by a few fold.

*Chain-Initiator Design Considerations*. The analysis of the transport initiation mechanisms in N_3_C*n* revealed that whereas there is a dominant initiation pathway for shorter chains (mechanism C on the twisting band), other mechanisms are expected to contribute more efficiently for longer chains. A higher density of chain states for longer chains is expected to result in a more efficient wavepacket formation on the wagging band via mechanisms B and A. Such a switch is expected for chain lengths exceeding *n* ≈ 30 methylene units when at least two wagging state will be strongly coupled to ν_N=N_. The transport speed will change to that corresponding to a local  derivative to the wagging band at the ν_N=N_ frequency, which is ∼36 Å/ps (Table S2). A further increase in the transport speed is expected for even longer compounds with *n* > 40 units, with a larger contribution of a wavepacket on rocking states with a speed () of ca. 68 Å/ps (Table S2).

Note also that it is advantageous to use only a portion of the chain band for a wavepacket, as such portions often feature almost a linear relation between the frequency and the wavevector, as is the case for the wagging and rocking bands at the positions of ν_N=N_ and ν_N–C_, respectively. If a linear relation between the frequency and wavevector is satisfied (ω ≈ *q* + constant), then the wavepacket will propagate without much broadening, similar to the propagation of a short laser pulse in a vacuum, ω = *cq*, where *c* is the speed of light in a vacuum. On the contrary, a wavepacket broadening is expected with time if the whole chain band is involved in the wavepacket.^28^

For 1D chains, chain-state localization is expected at some length, which will limit the coherence length for the wavepacket and thus the ballistic transport distance.^29,30^ Such localization will occur more readily for the states of narrow bands featuring smaller coupling among unit cells. The chain-state localization depends on intramolecular fluctuations as well as on interactions with the solvent.^22^ Intramolecular fluctuations include slow conformational variations, such as antigauche variations and fast thermal fluctuations. For broad alkane chain bands, such as wagging and rocking, the chain width exceeds by many fold the width of the inhomogeneous chain-site frequency distribution, which increases the length for chain-state localization. Further studies with longer compounds are required to understand the extent of the localization.

## 5 Conclusions

Different mechanisms of ballistic energy-transport initiation in oligomeric molecular chains are discussed and tested for transport initiation with ν_N≡N_ excitation in ^14^N_3_C*n*-a and ^15^N_3_C*n*-a compounds. We concluded that the dominant mechanism of wavepacket formation in relatively short compounds with *n* = 5, 10, and 15 involves a two-step relaxation into the twisting band states (mechanism C). Whereas the wavepacket via twisting band dominates the transport, multiple wavepackets involving different optical chain bands and transport speeds are found for both ^14^N_3_C*n*-a and ^15^N_3_C*n*-a compounds. The difference in the speed for the two isotopes is linked to a larger contribution of fast wavepackets for ^14^N_3_C*n*-a.

We concluded that the density of chain states in the studied chains (*n* ≤ 15) is insufficient to induce efficient wavepackets at the wagging and rocking bands via mechanisms A and B. These mechanisms may become dominant for longer chains offering significantly faster speeds of ca. 36 Å/ps via wagging and ca. 60 Å/ps via rocking bands. The study provides tools for analyzing ballistic transport initiation mechanisms in different molecular systems, which can lead to the design of systems featuring efficient initiation and efficient through-chain transport.

The ballistic transport was initiated in this study with mid-IR photon absorption; however, if ν_N≡N_ is excited by other means, such as thermally or as a result of electronic relaxation, its relaxation will initiate the same wavepackets in the chain as described here, rapidly transporting a significant portion of its energy to large distances along the alkane chain. Optimization of the energy injection and transport efficiencies can help in developing novel materials suitable for energy management and materials for molecular electronics.

## Supporting Information Available

Additional cross-peak data and details of their treatment, analysis of the FRs, dispersion curves for alkyl chains, lifetime data for ν_N≡N_, and vibrational relaxation pathways for ν_N–C_ (PDF)
