# Supplementary material for: Competition of Several Energy-Transport Initiation Mechanisms Defines the Ballistic Transport Speed
Source: J Phys Chem B. 2021 Jun 29;125(27):7546–55. doi: 10.1021/acs.jpcb.1c03986 (PMC8287563; doi:10.1021/acs.jpcb.1c03986)
Supplement: Supplementary file 1 — jp1c03986_si_001.pdf [file jp1c03986_si_001.pdf]

Supporting Information

A Competition of Several Energy Transport Initiation Mechanisms Defines the Ballistic Transport Speed

Sithara U. Nawagamuwage, Layla N. Qasim, Xiao Zhou, Tammy X. Leong, Igor V. Parshin, Janarthanan Jayawickramarajah, Alexander L. Burin, Igor V. Rubtsov*

Department of Chemistry, Tulane University, New Orleans, Louisiana 70118, United States
*corresponding author: irubtsov@tulane.edu

Table of contents

S1. Frequency shift by the isotope editing ($^{14}N_3$- → $^{15}N_3$)
S2. Additional 2DIR data and analysis details
S3. Calculating mean group velocity
S4. Atomic displacements of the low-frequency modes involved in $\nu_{N=N}$ relaxation
S5. Fermi resonances computed of $\nu_{N\equiv N}$
S6. Rocking chain band using anharmonic frequencies
S7. Relaxation pathways of $\nu_{N=N}$
S8. Lifetime measurements for $\nu_{N\equiv N}$
S9. Details of the classical modeling of ballistic transport.

S1. Frequency shift by the isotope editing ($^{14}N_3$ → $^{15}N_3$)

Significant shifts in frequency for the tag, $\nu_{N\equiv N}$, and its relaxation daughter mode, $\nu_{N=N}$, are observed consequent to isotopic substitution. Experimental (FTIR) and DFT calculated frequencies for $^{14}N_3C5$ and $^{15}N_3C5$ are shown in Table S1.

Table S1. Experimental and calculated frequencies of $\nu_{N\equiv N}$ and $\nu_{N=N}$

Mode		Frequency (cm^{-1})	
		$^{14}N_3C5$	$^{15}N_3C5$
$\nu_{N\equiv N}$	Experimental	2100	2031
	Cal.(harmonic)	2206	2132
	Cal.(anharmonic)	2136	2065
$\nu_{N=N}$	Experimental	1270	1225
	Cal.(harmonic)	1331	1287
	Cal.(anharmonic)	1303	1239

S2. Additional 2DIR data and analysis details

The $\nu_{N\equiv N}$ / $\nu_{C=O}$ cross peak spectra were measured for $^{15}N_3Cn$-a where n = 5, 10, 15 as a function of waiting time and the cross-peak amplitude kinetics were obtained. Figure S1 shows three waiting time dependences for each chain length, separately fitted with an asymmetric double sigmoidal function:

$$y = y_0 + A\left(\frac{1}{1+e^{-(x-xc+\frac{w1}{2})/w2}}\right)\left(1-\frac{1}{1+e^{-(x-xc+\frac{w1}{2})/w3}}\right) \qquad \text{(S1)}$$

Here y_0 is the offset, A is the amplitude, xc is the center of the peak, $w1$ is the full width of half maximum, $w2$ is the variance of low energy side, and $w3$ is the variance of high energy side.

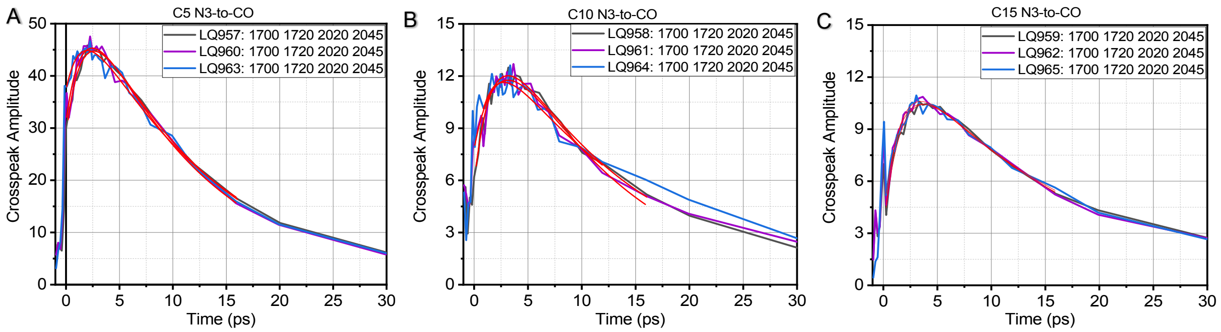

Figure S1. Waiting time traces of the $^{15}N_3$ / C=O cross peak amplitude for (A) $^{15}N_3$C5-a, (B) $^{15}N_3$C10-a, (A) $^{15}N_3$C15-a. The traces for three different experiments are shown for each chain length. The data were fitted with an asymmetric double sigmoidal function (red lines), Eq. S1.

S3. Calculating mean group velocity.

The ν(N=N) mode frequency falls within two chain bands, corresponding to the CH_2 wagging and twisting motions. Wagging and twisting dispersion curves computed for an alkyl chain are shown in Figure S2. A model of a linear chain of coupled states results in dispersion relations described by Eq. S2.

$$\omega(q) = \omega_0 + 2\beta \cos(qa) + 2\gamma \cos(2qa) \qquad \text{S2}$$

where ω_0 is the site energy, β is the nearest neighbor interaction, γ is the next nearest neighbor interaction, q is the wavevector and a is the lattice period. Equation S2 can be used to approximate actual dispersion curves for linear alkane chains. Detailed description of chain band construction is given elsewhere.[1]

The mean group velocity supported by the whole band was computed as: $\langle V_{\text{gr}} \rangle = \frac{a}{\pi}\int_0^{\pi/a} \frac{\partial \omega}{\partial q} dq$. If a small portion of a band at $\omega_{\text{end-gr.}}$ is involved in the wavepacket, covering a wavevector range from q_1 to q_2, the group velocity can be computed as $\langle V_{\text{gr}} \rangle = \frac{1}{(q_2 - q_1)}\int_{q_1}^{q_2} \frac{\partial \omega}{\partial q} dq$. If the portion of the band involved in the wavepacket is very narrow the mean group velocity of such wavepacket can be approximated as a point derivative to the dispersion curve at $\omega_{\text{end-gr.}}$: $< V_{\text{gr}} > \sim \left.\frac{\partial \omega}{\partial q}\right|_{\omega_{\text{end-gr.}}}$.

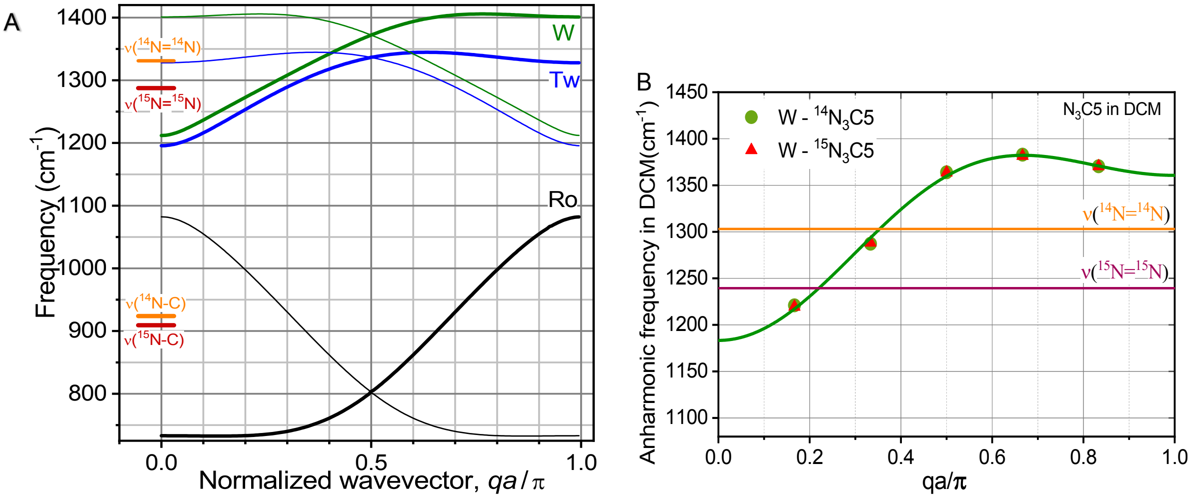

Figure S2. A. Dispersion curves for CH_2 twisting, wagging, and rocking bands of an alkane chain. Harmonic end-group frequencies ν(^{14}N=^{14}N), ν(^{15}N=^{15}N), ν(^{14}N-C), ν(^{15}N-C) are also shown. **B**. CH_2 wagging band constructed from DFT anharmonic calculations of $^{14}N_3$C5 and $^{15}N_3$C5. Anharmonic $\nu_{N=N}$ frequencies for both isotopes are shown.

Table S2. Group velocities for whole band, $\langle V_{\mathrm{gr}} \rangle$, and at indicated positions of a band.

Group velocity	Wagging band	Twisting band
$\frac{\partial\omega}{\partial q}$ Velocity at $\nu(^{14}N{=}^{14}N)$, (Å/ps)	35.9	21.3 / 9.1
$\frac{\partial\omega}{\partial q}$ Velocity at $\nu(^{15}N{=}^{15}N)$, (Å/ps)	36.0	32.7
Mean velocity, $\langle V_{\mathrm{gr}} \rangle$, for the whole chain band, (Å/ps)	15.9	10.4

Group velocity	Rocking band
$\frac{\partial\omega}{\partial q}$ Velocity at $\nu(^{14}N\text{-}C)$, (Å/ps)	67.6
$\frac{\partial\omega}{\partial q}$ Velocity at $\nu(^{15}N\text{-}C)$, (Å/ps)	67.5
Mean velocity, $\langle V_{\mathrm{gr}} \rangle$, for the whole chain band, (Å/ps)	30.8

As apparent from Figure S2 and Table S2, the point derivatives $\frac{\partial\omega}{\partial q}$ to both wagging and twisting band curves are significantly larger at the position of $\nu(^{15}N{=}^{15}N)$ compared to those at $\nu(^{14}N{=}^{14}N)$. Therefore, before performing the experiment, we expected that the red frequency shift of $\nu_{N=N}$ by isotopic substitution would lead to a higher transport speed in $^{15}N_3Cn$-a but opposite behavior was observed.

In an effort to explain experimentally observed speeds, molecule specific anharmonic effects on the wagging chain bands were considered. Wagging bands were constructed using DFT anharmonic calculations for the N_3C5 compounds (Fig. S2B). Chain modes showing wagging motion type were grouped into a band and fitted with Eq. S2. A wavevector for each chain state was identified by the number of the nodes for the delocalized chain-state wavefunction. The wavevector for mode *i* was computed as $q_i = \frac{\pi}{(n+1)a}(N_i + 1)$, where *n* is the number of methylene groups in the chain, *a* is the unit-cell length, computed at 1.53 Å, and N_i is the number of nodes. As expected, the wagging band is not affected by the end-group isotope editing (Fig. S2B). The bandwidth obtained with anharmonic state frequencies appeared to be wider than that for harmonic states by ca. 15%. As a result of the band changes, the transport speed obtained as $< V_{\mathrm{gr}} > \sim \left.\frac{\partial\omega}{\partial q}\right|_{\omega_{\mathrm{end-gr.}}}$ appears to be higher than that for harmonically determined band, at 43 and 41 Å/ps for $^{14}N_3C5$ and $^{15}N_3C5$, respectively.

S4. Atomic displacements of the low-frequency modes involved in $\nu_{N=N}$ relaxation

Intramolecular vibrational energy redistribution (IVR) is an essential process for the thermalization of a molecule. This process is often described via anharmonic interactions of normal modes in the molecule. A large portion of relaxation pathways of $\nu_{N=N}$ (*i*) involve combination states of one of the twisting modes (*j*) and a low frequency mode (*k*). The low frequency modes (*k*) involved in such relaxation are shown in Table S3.

Table S3. Low-frequency mode (*k*) involved in vibrational relaxation of $\nu_{N=N}$ in N_3C5 for both isotopes.

k mode number	Frequency (cm^{-1})	Vector displacements of atoms for each mode
45	148	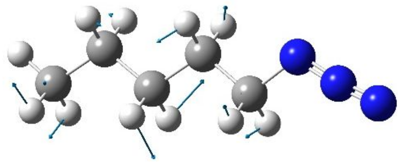

46	135	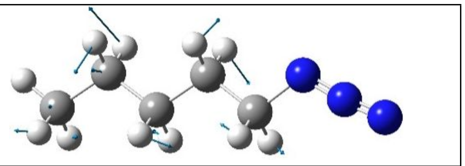
48	81	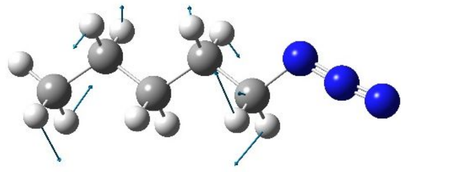
49	60	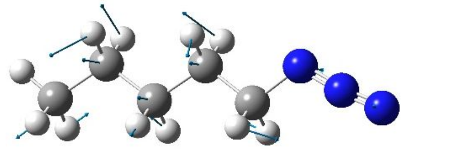
51	25	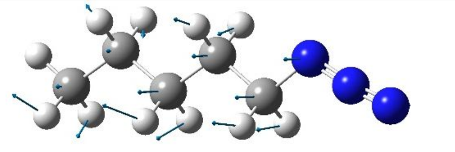

S5. Fermi resonances computed of $\nu_{N\equiv N}$

The effect of CNN angle variation and Fermi resonance on the $\nu_{14N\equiv N}$ transition has been studied elsewhere for $^{14}N_3C3$.[2] Here we performed DFT calculations of Fermi resonances for $^{15}N_3C3$ and found the presence of multiple Fermi resonances. Although Fermi resonances were found for a wide range of CNN angles for $^{15}N_3C3$ (Fig. S3B), their number is smaller than that for $^{14}N_3C3$ (Fig. S3A). It is conceivable that Fermi resonances contribute to the smaller T_{max} values observed for $^{14}N_3Cn$-a compounds.

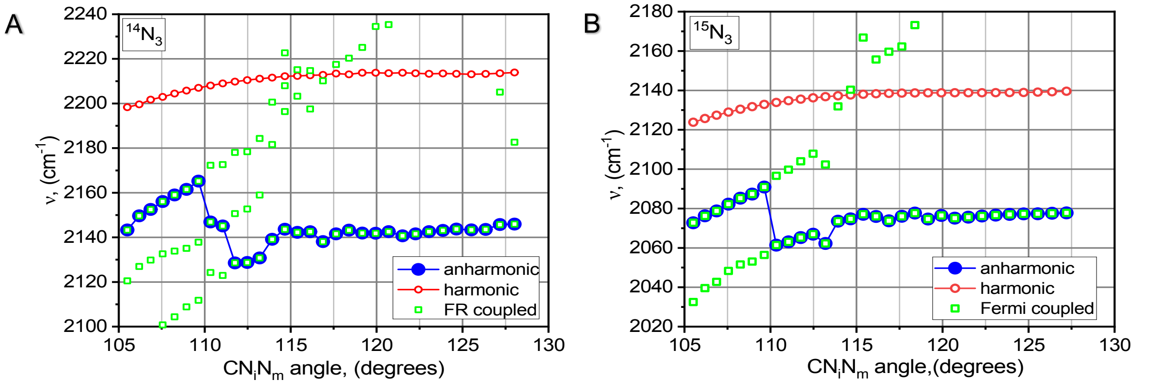

Figure S3. CNN angle dependence of frequency and Fermi resonance for $\nu_{N\equiv N}$ for (A) $^{14}N_3C3$ and (B) $^{15}N_3C3$. Harmonic (red), anharmonic reported by Gaussian (blue), and all Fermi-coupled anharmonic states (green) are indicated.

S6. Rocking chain band using anharmonic frequencies

Mixing of the $\nu_{N\text{-}C}$ mode with the rocking band states is negligible, so the rocking band does not change upon $^{14}N_3 \rightarrow {}^{15}N_3$ end-group substitution. Figure S4 shows that the changes of the rocking band are not very large if anharmonic rocking states are considered.

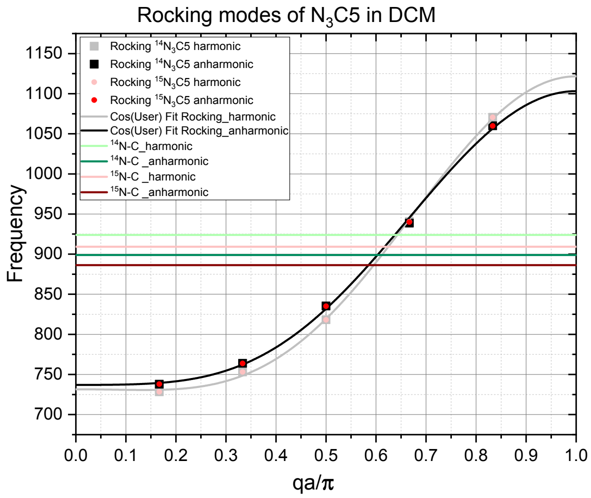

Figure S4. CH_2 rocking band constructed from DFT harmonic and anharmonic calculations of $^{14}N_3C5$ and $^{15}N_3C5$. Harmonic and anharmonic $\nu_{N\text{-}C}$ frequencies for both isotopes are shown.

S7. Relaxation pathways of $\nu_{N=N}$

Analysis of the IVR relaxation pathways for $\nu_{N=N}$ revealed that many bands receive energy with a significant rate contribution. All relaxation pathways of $\nu_{N=N}$ for N_3C5 in DCM is shown below. Both isotopes prominently relax to the twisting band states, N-C stretch mode and an overtone of the NNN bending mode.

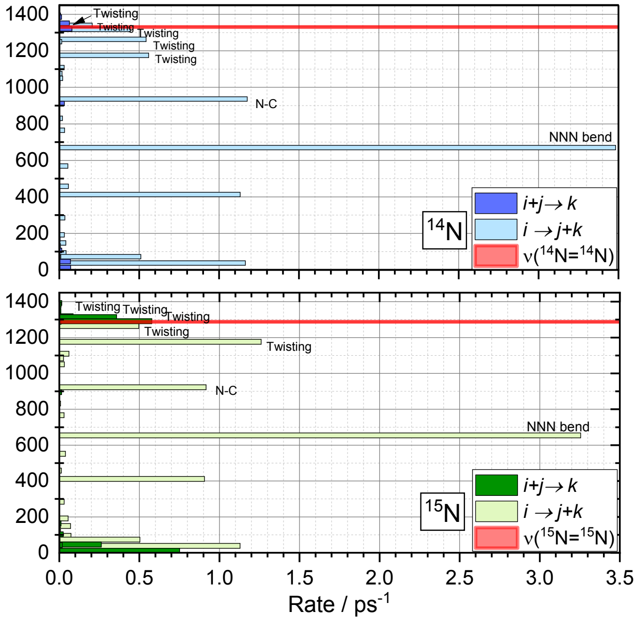

Figure S5. IVR relaxation pathways and rates for $\nu_{N=N}$ for both isotopes (^{14}N - blue and ^{15}N - green). The $\nu_{N=N}$ frequency is marked (red line).

S8. Lifetime measurements for $\nu_{N\equiv N}$

Diagonal $\nu_{N\equiv N}$ peaks for $^{15}N_3C5$-a were measured as a function of the waiting time (Fig. S5A), integrated, and plotted as a function of the waiting time (Fig. S5B). The trace was fitted with a two-exponential function (red line in Fig. S5B) resulting in decay times of 0.84 ± 0.05 ps (77% amplitude) and

7.0 ± 1.1 ps (23% amplitude). The dominant fast decay component is associated with the $\nu_{N\equiv N}$ lifetime, while the second component is likely associated with cooling of the excess energy at the azido group. The $\tau_{1/e}$ value of 1.4 ps was obtained for $^{15}N_3C5$-a. For comparison, the $\tau_{1/e}$ value of 1.47 ps was obtained for $^{14}N_3C5$-a, indicating that the $\nu_{N\equiv N}$ lifetimes for both isotopes do not differ much.

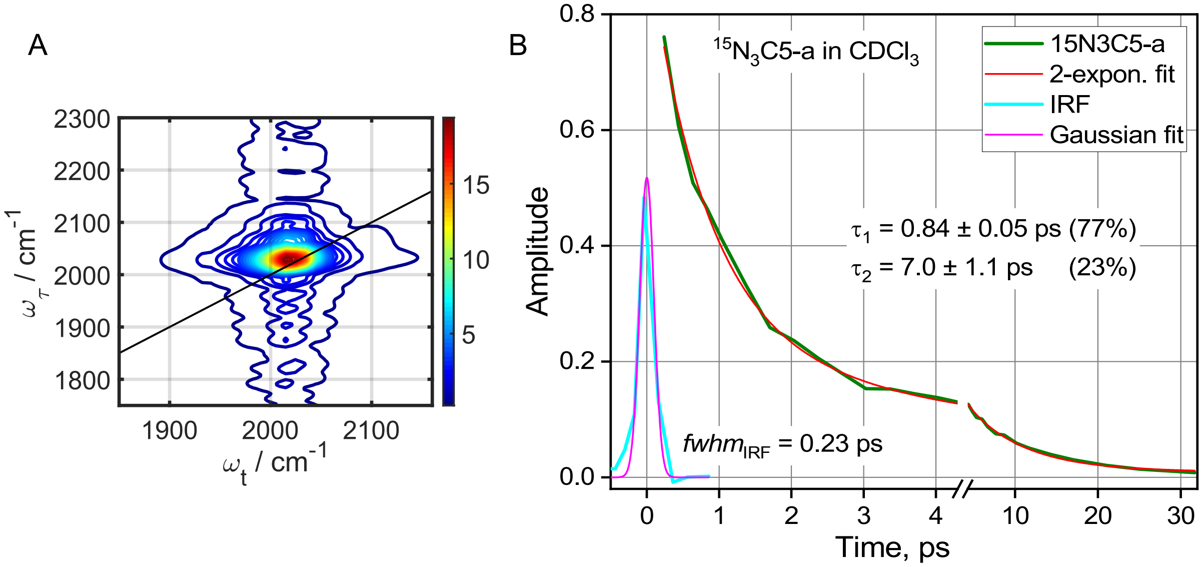

Figure S6. A. Magnitude $\nu_{N\equiv N}$ diagonal peak for $^{15}N_3C5$-a measured at 0.55 ps delay. **B.** Waiting time trace of the diagonal peak amplitude (green line) fitted with a two-exponential function (red line). Instrument response function is also shown (cyan line) fitted with a Gaussian profile (magenta line).

S9. Details of the classical modeling of ballistic transport. Ballistic energy transport in N_3Cn-a was modeled by solving classical Newton's equations using DFT-determined harmonic force constants (Hessian). Note that harmonic approximation is expected to work reasonably well for the wavepacket transport. The initial excitation at ca. 1300 cm^{-1} was introduced by exciting a local $\nu_{N=N}$ mode. This local mode has been identified as the second highest vibrational frequency of the azido group, obtained by diagonalizing the reduced Hessian matrix (the part of the Hessian matrix associated with the three nitrogen atoms only). Initial displacements for nitrogen atoms have been set proportional to corresponding eigenvector components, while other displacements and all momenta have been set equal to zero at the initial time t = 0.

Energy arrival to the reporter was monitored by summing all local energies of atoms belonging to the COOH end group. The local energies are defined for each atom i and each Cartesian coordinate x as

$$h_{i\alpha} = p_{i\alpha}{}^2/2m_i + 1/2\sum_{j\beta}(H_{ij\alpha\beta}x_{i\alpha})x_{j\beta},$$

where H stands for the Hessian matrix and p are the momenta. The analysis of the end-group energy time evolution was used to estimate the ballistic transport time and velocity as described below.

The time dependence of the COOH end-group energy was evaluated solving Newton's equations employing MATLAB software. The same Hessian matrix was used for normal and isotopically modified samples and the difference is originated only from the mass difference.

Representative time dependencies of the end-group energy are shown in Figure S7 for the normal and isotopically modified N_3C5-s samples at delay times between 0 and 10 ps. It turns out that these dependences are periodic. Consequently, one can estimate the transport time assuming that the period represents the double travel time of the energy along the chain.

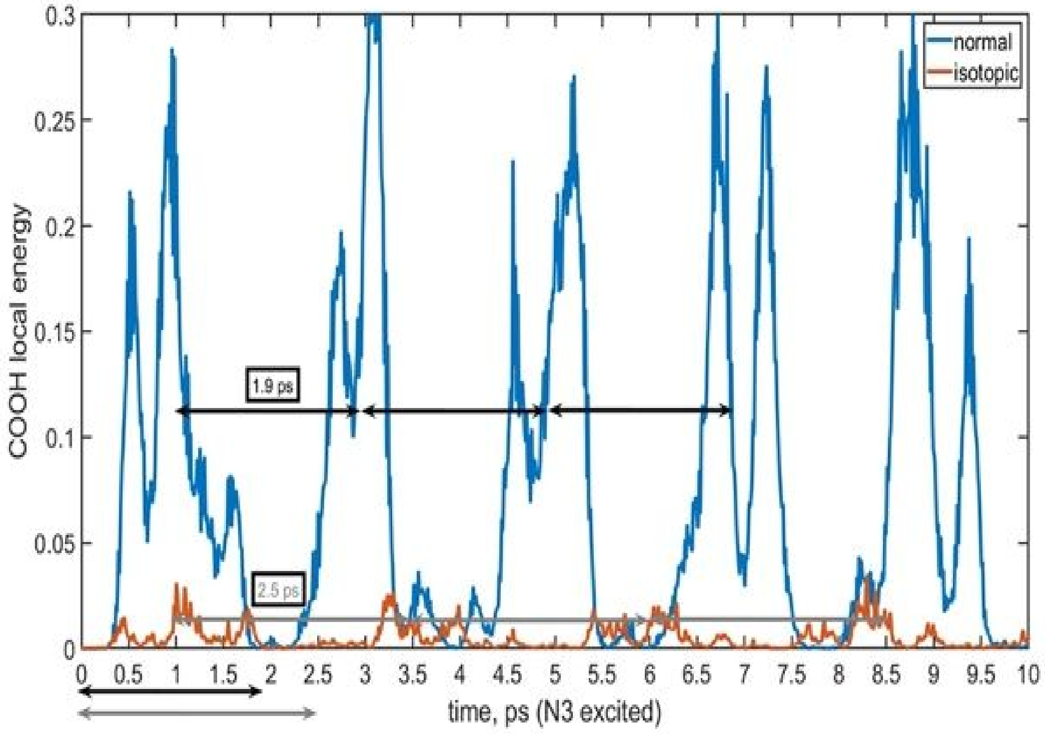

Figure S7. COOH end-group energy vs. time for the normal and isotopically edited N_3C5-s compounds. Energy is expressed in arbitrarily units.

The period, t_R, of the recurrency peaks was determined, as indicated in Figure S7 by double-sided arrows for normal and isotope samples. The energy transport velocity was determined independently for compounds with every chain length, *n* (*n* = 5, 10, 15) as

$$V(n) = 2 \cdot \frac{n \cdot l_{CC} + l_{CN}}{t_R} \quad \text{(S3)}$$

where $V(n)$ is the transport velocity, t_R is the transport period, and l_{CC} (1.54 Å) and l_{CN} (1.48 Å) are the C-C and C-N bond lengths, respectively. The results for all samples are summarized in Table S4.

Table S4. Transport velocities calculated with Eq. S3 for normal and isotopically substituted N_3C*n*-a compounds with *n* = 5, 10, 15. No energy damping was introduced.

	$^{14}N_3C$*n*-a		$^{15}N_3C$*n*-a	
n	Period, t_R (ps)	Velocity, *V* (Å/ps)	Period, t_R (ps)	Velocity, *V* (Å/ps)
5	1.9	9.7	2.5	7.3
10	2.3	14.7	5.0	6.8
15	5.0	9.8	9.0	5.5

Alternatively, one can investigate the transport time considering the arrival time in the presence of energy damping, which represents relaxation and/or decoherence of the chain states. Following previous work, we set the damping time to be τ = 1.5 ps and incorporated it into Newton's equations for momenta via $-p/\tau$ terms. After solving the Newton's equations, one obtains that the COOH excess energy, $E(t)$, decays rapidly with the time increase (Fig. 7C of the main text). To estimate the transport time, we used the average arrival time, t_D weighted with the function $E(t)$ as

$$t_{\mathrm{D}} = \int_0^{\infty} E(t) t dt \quad \text{(S4)}$$

With this definition for t_{D}, the transport velocity can be estimated as

$$V = \frac{n \cdot l_{CC} + l_{CN}}{t_{\mathrm{D}}}. \quad \text{(S5)}$$

The estimates of transport velocity based on arrival times are shown below in Table S2.

Table S5. Transport velocities calculated using Eq. S5 for normal and isotopic N_3C*n*-a compounds with *n* = 5, 10, 15 with the damping factor of 1.5 ps^{-1}.

	$^{14}N_3C$*n*-a		$^{15}N_3C$*n*-a	
n	t_{D} (ps)	Velocity (Å/ps)	t_{D} (ps)	Velocity (Å/ps)
5	0.8	11.5	1.0	9.1
10	1.2	14.1	1.8	9.3
15	1.7	14.5	2.3	10.7

The normal mode analysis of the initial excitation suggests that CH_2 wagging modes in the region 1330 – 1360 cm^{-1} are strongly involved in the energy transport; the resulting velocities are different for the two isotopes and slightly depends on the length of the molecule. These results are used in the main text to discuss the origin of the isotope effect in the ballistic transport.

References

1. Yue, Y.; Qasim, L. N.; Kurnosov, A. A.; Rubtsova, N. I.; Mackin, R. T.; Zhang, H.; Zhang, B.; Zhou, X.; Jayawickramarajah, J.; Burin, A. L.; Rubtsov, I. V., Band-selective ballistic energy transport in alkane oligomers: toward controlling the transport speed. *J. Phys. Chem. B* **2015,** *119* (21), 6448-56.
2. Varner, C.; Zhou, X.; Saxman, Z. K.; Leger, J. D.; Jayawickramarajah, J.; Rubtsov, I. V., Azido alkanes as convenient reporters for mobility within lipid membranes. *Chem. Phys.* **2018,** *512*, 20-26.
